# Wear and Breakage Detection of Integral Spiral End Milling Cutters Based on Machine Vision

**DOI:** 10.3390/ma14195690

**Published:** 2021-09-30

**Authors:** Wenming Wei, Jia Yin, Jun Zhang, Huijie Zhang, Zhuangzhuang Lu

**Affiliations:** State Key Laboratory for Manufacturing Systems Engineering, School of Mechanical Engineering, Xi’an Jiaotong University, Xi’an 710049, China; wm_wei@xjtu.edu.cn (W.W.); Yinjia6@163.com (J.Y.); zhanghuijie@xjtu.edu.cn (H.Z.); zhzh_lu@163.com (Z.L.)

**Keywords:** integral spiral end milling cutter, wear and breakage detection, machine vision, image processing

## Abstract

Tool wear and breakage detection technologies are of vital importance for the development of automatic machining systems and improvement in machining quality and efficiency. The monitoring of integral spiral end milling cutters, however, has rarely been investigated due to their complex structures. In this paper, an image acquisition system and image processing methods are developed for the wear and breakage detection of milling cutters based on machine vision. The image acquisition system is composed of three light sources and two cameras mounted on a moving frame, which renders the system applicable in cutters of different dimensions and shapes. The images captured by the acquisition system are then preprocessed with denoising and contrast enhancing operations. The failure regions on the rake face, flank face and tool tip of the cutter are extracted with the Otsu thresholding method and the Markov Random Field image segmentation method afterwards. Eventually, the feasibility of the proposed image acquisition system and image processing methods is demonstrated through an experiment of titanium alloy machining. The proposed image acquisition system and image processing methods not only provide high quality detection of the integral spiral end milling cutter but can also be easily converted to detect other cutting systems with complex structures.

## 1. Introduction

Tool wear and breakage have a great effect on the quality of machined components and production efficiency. Lacking efficient detection methods of tool failure forms will lead to facture, large tolerance, and even damage of machine tools, resulting in great economic loss. Machine tools equipped with a tool condition detection system are found to have their downtime reduced by 75%, production efficiency enlarged by 10% to 60% and utilization ratio improved by more than 50% [1,2,3]. The rapid development of intelligent machining and advanced machining technologies in recent years also dramatically increases the demand for advanced detective technologies of tool failure forms.

The present tool failure form detection technologies fall into two categories: indirect and direct methods. The indirect methods determine the tool failure forms through the analysis of various signals generated in different cutting conditions (such as acoustic emission [4,5], temperature variation [6], strain [7], vibration signals [8,9,10], motor current [11,12], power of spindle [13] and cutting forces [14]), it is thus required that a great deal of high performance sensors are installed at the specified locations of the machining equipment, which significantly increases the machining costs as well as producing obstacles in the working spaces [15]. The direct methods evaluate the tool failure forms by a machine vision method. The cutting zone shape variation can be measured with cameras, microscopes, or scanners; the wear and breakage can then be extracted by image processing techniques with captured images [16,17]. Machine vision has been successfully applied to analyze tool failure forms by examining tool surfaces, machined workpiece surfaces, and chip morphology. The examination of tool surfaces is the most straightforward method in detecting the tool failure forms, hence it has been widely adopted in the published papers. For instances, Ong et al. [18] found that the tool wear degree could be predicted more accurately under different cutting parameters and machine time with wavelet neural network. Wang et al. [19,20] studied the wear and breakage of different inserts of rotated milling cutters by capturing dynamic images and processing them with a rough-to-fine strategy and threshold independent edge detection method. Ryabov et al. [21] reconstructed the 3D tool images of inserts by laser scanners to determine the flank wear length of worn tool. The tool failure forms can affect the surface textures of workpieces; therefore, the examination of machined workpiece surfaces has been employed for the detection of tool failure forms. For instance, Kassim et al. [22] found it reliable to obtain the sharp, semi-dull and dull status of cemented carbide inserts by analyzing machined surface textures with run-length statistical method. Yu et al. [23] evaluated the tool wear with the achieved fractal dimensions of workpiece local images. Li et al. [24] proposed a multi-feature information synthesis method which could estimate the tool failure with both the workpiece texture and tool wear images. In addition, the chip morphology is related to the tool failure; hence, it has also been used to detect the tool failure forms. Yuan et al. [25] developed a feature-expanding method and an artificial neural network recognition algorithm to analyze cutter wear and breakage by chip morphology recognition. Zhang et al. [26] found that the wear region dimensions of worn inserts could be predicted by the width and radius of the chip morphology. Pagani et al. [27] presented a deep learning approach to monitor tool wear by different indicators, extracted from colorful images of turning chips. Direct detection technologies based on machine vision demonstrate great advantages over indirect technologies due to the minor effects on the machining system, check intended meaning retained. The cost of the direct detection technologies is also lower than that of indirect methods. Firstly, the failure is detected with several cameras for the direct machine vision methods instead of a great deal of high-performance sensors; thus, the hardware cost are lowered. Besides, the machine vision system can be easily mounted onto the machine tools and the machine tool will not need to be reconfigured to accommodate many sensors, therefore the installation cost can be reduced. In addition, the maintenance cost of the machine vision system with several cameras is also lower than that of multiple sensors. Direct detection technologies therefore possess significant potential for widespread industrial applications, including optical coordinate measuring machine for dimensions and form measurement of workpiece, 3D optical profiler for surface texture examination, tool inspection machine for tool geometry and wear measurement, and defect detection for quality control, etc.

Integral spiral end milling cutters are widely used in the contouring milling of a complex workpiece. Numerous direct machine vision methods have been proposed to detect the failure forms of various machined tools, including turning tools [28], broaching tools [29], drill bit [30], grinding wheels [31] and inserts for turning [32] and milling [19,20,33], etc. Whereas these methods could rarely be applied to detect the failure regions on integral spiral end milling cutters due to their complex structures. Besides, the images of different regions on the tools are often separately captured because of their varied geometries and locations, which will require multiple image acquisition systems and reduce the efficiency of acquisition. It is thus critical to develop an image acquisition system and image processing methods which can provide reliable detection for integral spiral end milling cutters.

In this paper, a tool wear and breakage detection method is proposed for the first time in order to analyze the failure transformation of integral spiral end milling cutters during machining. A flexible image acquisition system with adjustable light sources is developed to capture the images of machined surfaces on milling cutters of various dimensions. The failure regions are identified from the captured images with specific segmentation methods to evaluate the wear and breakage on rake face, flank face and tool tip. The feasibility of the proposed methods is verified experimentally with the machining of titanium alloy. It should be noted that the system captures images when the machining process is paused. Compared with simple optical control of the cutting surface, the proposed machine vision system requires extra hardware and software to capture and analyze images. However, the system has non-negligible and irreplaceable advantages over simple optical control in these aspects: firstly, although the proposed system deals with still pictures, it has an automated process; the machining stops at the preset time, then images are captured and sent for analysis, after that the machining can restart. The above mentioned procedures may repeat multiple times. While the simple optical control method may require taking the cutters off from the machine for checking and failure measuring, the machining process is hard to restart. In addition, the proposed quantitively system can detect the failure regions, and the output pixel numbers can clearly indicate the status of the cutter and whether replacement is needed. Conversely, the simple optical method can only provide a qualitative estimation of the failure on the cutters, and additional precision instruments such as microscopes are required for the measurement of actual failure regions. Moreover, the proposed system could be upgraded to realize real-time detection with corresponding instruments such as high-speed cameras, high performance computers, etc. The main challenges facing the proposed method are robustness and accuracy improvement with advanced algorithms and equipment, and errors estimation at different machining environment, etc.

The paper is structured as follows: Section 2 depicts the image acquisition system and image preprocessing procedures; Section 3 shows the failure extraction methods for the different parts of the cutters; Section 4 describes the experimental verification of the proposed systems and methods; Section 5 includes the conclusions of the paper.

## 2. Image Acquisition System and Preprocessing

This section introduces the proposed adjustable image acquisition system and its working mechanisms. The images captured by the system must be preprocessed before they can be used for the failure extraction, the prepossessing procedures and algorithms are then explained.

### 2.1. Design of Adjustable Image Acquisition System

**Figure 1:**
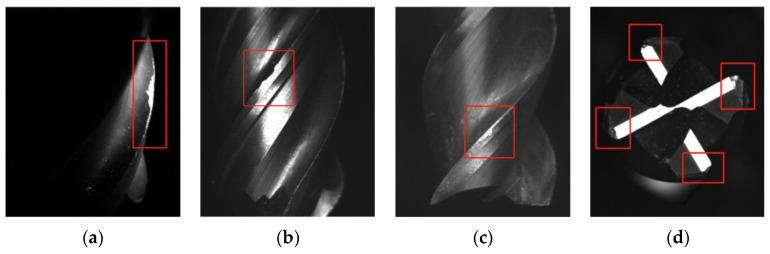
Photos of typical failure forms of integral spiral end milling cutter: (**a**) Crater wear; (**b**) Flank wear; (**c**) Edge chipping; (**d**) Tool tip breakage.

For the integral spiral end milling cutter, rake face and flank face of principal, auxiliary cutting edge, and tool tip are all involved in the cutting process. The cutter tends to fail due to the combination of various wear forms, such as mechanical wear, abrasive wear, diffusion wear, oxidative wear, and breakage under large cutting forces and huge amount of heat. In general, crater wear, flank wear, edge chipping and tool tip breakage are the main failure forms of end mills, as shown in Figure 1.

The cutting portion of the milling cutters contains spatial curved surfaces. It is difficult to capture high-quality images of the spatial curved surfaces with ordinary methods due to severe image overlap and interference. It is therefore necessary to design specific image acquisition methods for different parts of the integral spiral end milling cutters.

**Figure 2:**
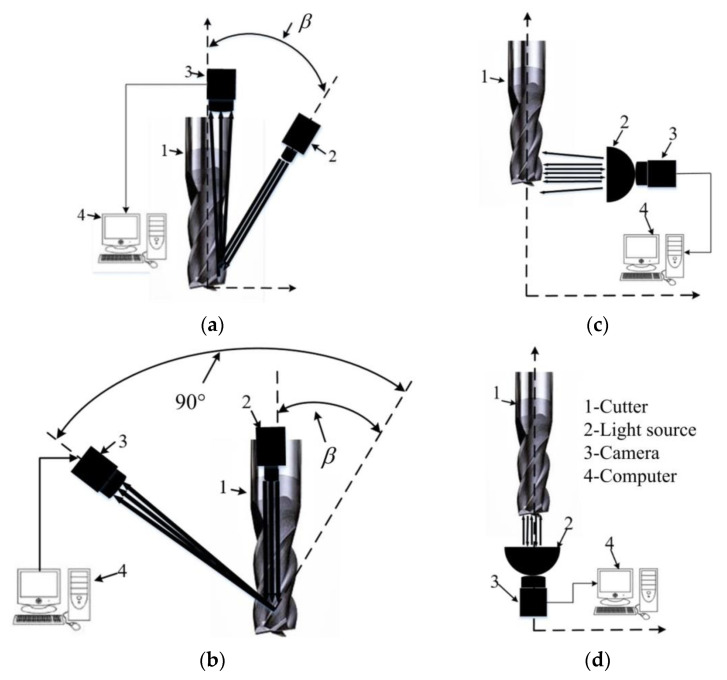
Schematic diagrams of image acquisition methods for different parts: (**a**) Side view of rake face with point light and cameras; (**b**) Back view of rake face with point light and cameras; (**c**) Flank face with dome light and cameras; (**d**) Tool tip with dome light and cameras. β is the helix angle of the cutter.

The proposed image acquisition system is schematically plotted in Figure 2. The targeted capture regions on the cutter firstly need to be illuminated for better image capture quality. A lighting system composed of a point and two dome light sources is designed for the integral spiral end milling cutter. The point light source (Figure 2a,b) is used for the rake face because of the small spot area and the easy adjustment of the spatial position; the two dome light sources are employed for the flank face (Figure 2c) and tool tip (Figure 2d) due to their large area and better light reflection. The angle between the point light source and tool axis is set equal to β, where β is the helix angle of the cutter as shown in Figure 2a,b. The dome light source for the flank face is perpendicular to the tool axis and the other dome light source for the tool tip parallel to the tool axis as shown in Figure 2c,d. The images of tool surfaces are then captured by two high-resolution cameras. Whilst one camera is employed for the rake face and the inclination of the camera is set as (90°-β) as shown in Figure 2b, the other camera, used for the flank face and tool tip, lies on top of the dome light sources as shown in Figure 2c,d. The captured images are finally transferred into the computer for further processing.

**Figure 3:**
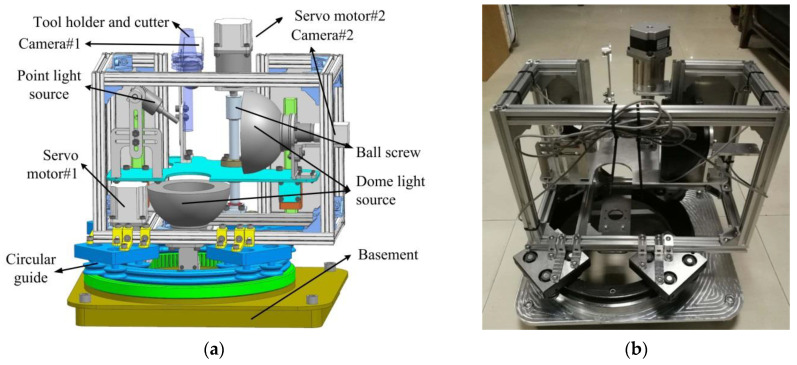
The proposed image acquisition system: (**a**) Structure components; (**b**) Assembled system.

The corresponding image acquisition system with all of the components is shown in Figure 3. A moving frame is installed on the square base through a ring rail and sliders, shown in Figure 3a. The movement of frame is driven by two servo motors. The light sources and cameras are fixed on the moving frame, their positions and postures could thus be easily adjusted to adapt to cutters with different diameters and lengths. All of the components are manufactured and assembled to build the acquisition system, as shown in Figure 3b. The camera is an acA2500-14gm GigE (Basler ace) area scan camera with five million pixels. It should be stressed that in case coolant is adopted in the machining process, a gas nozzle could be added to the system to introduce high pressure gas which can blow away the residual coolant before images are captured. Besides, a sealed enclosure could be designed for the image acquisition system to isolate the coolant during machining process.

### 2.2. Image Preprocessing

The quality of the captured images is restricted by the actual cutting environment, nonuniform light, and limited hardware performance; therefore, the raw captured images must go through a preprocessing before they can be applied for further failure extraction. The preprocessing includes denoising and contrast enhancing.

There are two main image noises in the captured images: Gaussian noise and impulse noises. The Gaussian noise is a statistical noise whose probability density function is equal to the normal distribution. The Gaussian noise occurs in low-light conditions, and the high temperature of sensors, etc. The impulse noise is is a result of the addition of random bright and dark by malfunctioning of the decoder, limited quality of imaging sensors, etc.

The Gaussian noise is generally eliminated by the mean filtering algorithm [34,35], i.e., replacing the pixel value with the average value of its neighbors, while the impulse noise is removed with the median filtering algorithm [36,37], i.e., replacing the pixel value with the median of neighboring values. The mean filtering method has the advantages of simplicity, intuitiveness, and easy implementation; however, it could cause problems such as edge blurring and unreliable mean values. The median filtering algorithm can tackle the problems of the mean filtering algorithm, although it requires longer computation time. A hybrid adaptive filtering method [38] is therefore used for the noise filtering. The two noises are first discriminated with the gray level of the central pixel in the filtering window and the mean value and local variance of the neighborhood, by

(1) $$W=\{\begin{array}{c} 1\left|I-\mu \right|\geq \sigma \\ 0\left|I-\mu \right|<\sigma \end{array}$$

where *I* is the gray level of the central pixel, μ and σ are the mean and local variance of neighboring values, respectively. After that, the noises are removed, respectively, by the two different filtering algorithms mentioned above.

**Figure 4:**
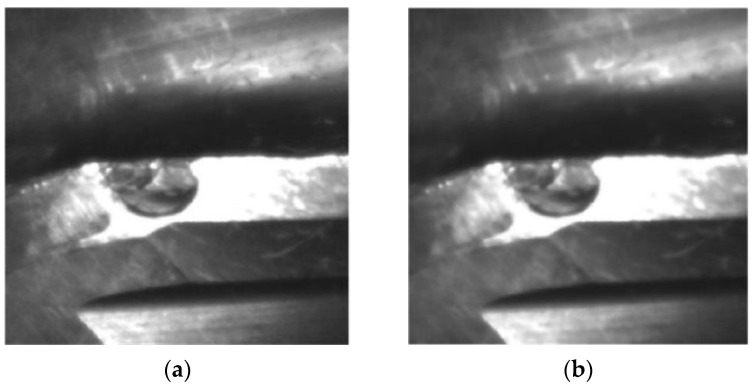
Comparison of flank face image before and after denoising: (**a**) Undenoised image; (**b**) Denoised image.

Figure 4 provides an example of a captured image of the flank wear of a ball end cutter before and after the denoising by the hybrid adaptive filtering. It should be noted that the edges are softened by the denoising algorithms, and the contrast enhancing is thus necessary for the images after denoising.

The image contrast represents the difference in luminance or gray values between the object with other objects or the background. Contrast enhancement can improve the quality of images by expanding the range of brightness values. Given the edge dulling in the images after the denoising, a logarithmic transformation method is employed to enhance the contrast of the images [34]. The gray level value f(r) for a given pixel r can be transformed by

(2) $$g\left(r\right)=a+\frac{\ln[f\left(r\right)+1]}{b\times \ln c}$$

where g(r)  is the gray level value after the transformation, a, b, c are adjustable empirical parameters.

**Figure 5:**
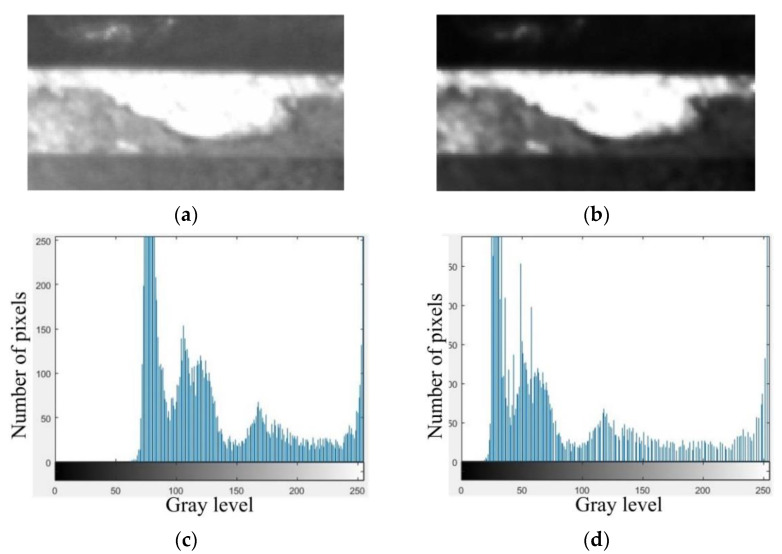
Images of flank wear before (**a**) and after (**b**) contrast enhancement. Gray level histograms that describe the number of pixels corresponding to all gray level values of images (**a**,**b**) are plotted in (**c**,**d**).

Images of a principal flank wear before and after the logarithmic transformation are shown Figure 5a,b. The enhanced image has a stronger bright and darkness contrast visually compared with the untransformed image, and the boundaries also become clearer. The gray level histograms which describe the number of pixels corresponding to all gray level values for Figure 5a,b are shown in Figure 5c,d. The range of gray level values is largely expanded as shown in Figure 5c,d.

## 3. Failure Region Extraction Methods

For the purpose of evaluating the wear and breakage status of the cutters, the failure region must be extracted from the images after the preprocessing procedures mentioned above. This section introduces the techniques involved for the failure region extraction of different parts of the integral spiral end milling cutters.

### 3.1. Extraction of Failure Region on the Flank Face and Tool Tip

The images of flank face and tool tip contain only background and the cutter body under the dome light sources. To extract the failure regions, the background and cutter body are first discriminated by image binarization using the Otsu thresholding method. Threshold techniques are widely used for image segmentation [39,40,41], the Otsu thresholding method is one of most popular threshold techniques that has attracted great attention due to its simple algorithm, high computational efficiency, and good segmentation effect [42,43,44]. The Otsu thresholding method searches for a threshold value that partitions the pixels into two classes based on the histograms of gray level, the threshold value can be obtained by maximizing the between-class variance.

Assuming the pixels of a given image can be represented in *L* gray levels {1,2,⋯,L}, the total number of pixels is *N*, the number of pixels at gray level *i* is denoted by $n_{i}$, the probability of the gray level *i* is $p_{i}=n_{i}/N$, where $p_{i}\geq 0$. A threshold value *k* can dichotomize the pixels into two classes: $C_{1}$ is composed of pixels with gray values of {1,2,⋯,k} and $C_{2}$ is composed of the pixels with gray values of {k+1,k+2,⋯,L}. The probabilities of pixels falling into the two different classes are

(3) $$P_{r1}\left(k\right)=\overset{k}{\underset{i=1}{\sum }}p_{i}$$

(4) $$P_{r2}(k)=\overset{L}{\underset{i=k+1}{\sum }}p_{i}=1-P_{r1}\left(k\right)$$

Furthermore, the average gray values of pixels in $C_{1}$ and $C_{2}$ are

(5) $$m_{1}\left(k\right)=\frac{1}{P_{r1}\left(k\right)}\overset{k}{\underset{i=1}{\sum }}ip_{i}=\frac{m\left(k\right)}{P_{r1}\left(k\right)}\;$$

(6) $$m_{2}\left(k\right)=\frac{1}{P_{r2}\left(k\right)}\sum_{i=k+1}^{L}ip_{i}=\frac{m_{G}-m\left(k\right)}{1-P_{r1}\left(k\right)}$$

where

(7) $$m\left(k\right)=\overset{k}{\underset{i=1}{\sum }}ip_{i}\;$$

(8) $$m_{G}=\overset{L}{\underset{i=1}{\sum }}ip_{i}$$

A criterion η(k) can be adopted to evaluate the binarization of threshold *k* [42]

(9) $$\eta \left(k\right)=\frac{\sigma_{B}^{2}\left(k\right)}{{\sigma_{G}}^{2}}\;$$

where $\sigma_{G}^{2}=\overset{L}{\underset{i=1}{\sum }}{\left(i-m_{G}\right)}^{2}p_{i}\;$ is the total variance, it remains constant for a given image. $\sigma_{B}^{2}\left(k\right)$ is the between-class variance which is given as

(10) $$\sigma_{B}^{2}=P_{r1}\left(k\right)P_{r2}(k){\left(m_{1}\left(k\right)-m_{2}\left(k\right)\right)}^{2}=\frac{{\left(m_{G}P_{r1}\left(k\right)-m\left(k\right)\right)}^{2}}{P_{r1}\left(k\right)\left(1-P_{r1}\left(k\right)\right)}\;$$

The optimal threshold $k^{*}$ can be obtained by

(11) $$\sigma_{B}^{2}\left(k^{*}\right)=\underset{1\leq k\leq L}{\max}\sigma_{B}^{2}\left(k\right)\;$$

**Figure 6:**
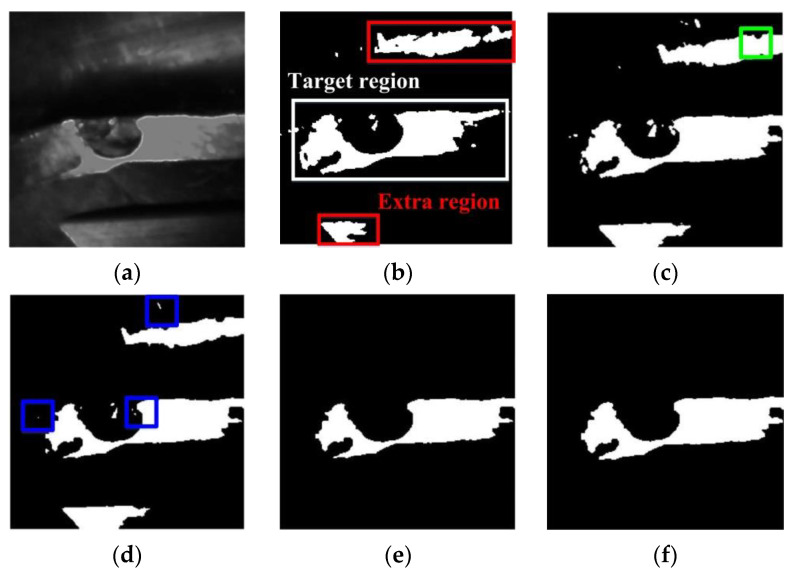
Segmentation and morphological operations of flank face image: (**a**) Preprocessed image; (**b**) Binarized image; (**c**) Hole filling; (**d**) Erosion; (**e**) Extraction of largest connected components; (**f**) Expansion.

The preprocessed and binarized images of a flank face are compared in Figure 6a,b. The image is segmented by the thresholding method. Besides, it can also be seen that, except from the cutter body (marked in the white box in Figure 6b), non-targeted regions (marked in red boxes in Figure 6b) exist in the binarized image, which would affect the failure extraction and increase the computing difficulty. The non-targeted regions can be removed with morphological operations of hole filling (Figure 6c), erosion (Figure 6d), extraction of largest connected components (Figure 6e) and expansion (Figure 6f).

**Figure 7:**
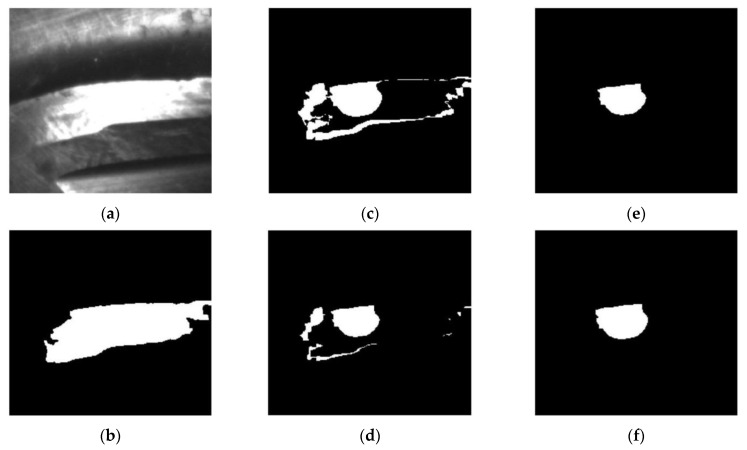
Wear region extraction of flank face image: (**a**) Unmachined flank face; (**b**) Processed unmachined flank face. Morphological operations of (**c**) subtracted, (**d**) erosion, (**e**) extraction of largest connected component and (**f**) expansion.

It should be mentioned that the reflected light of the wear region cannot be perceived by the cameras and that the wear regions on the cutter are concealed in the background region. The wear region needs to be extracted with the images of the cutter both before and after machining. Figure 7a shows the image of the flank face before machining, which is obtained via the same processing procedures as the image after machining. With the morphological operations of subtraction (Figure 7c), erosion (Figure 7d), extraction of largest connected components (Figure 7e) and expansion (Figure 7f), the wear region is extracted. The pixel number $S_{p}$ of the wear region is finally counted to evaluate the wear region and the cutter working status [3]. Similarly, the tool tip failure of the flat end milling cutter can be achieved following the same procedures of the flank face.

### 3.2. Extraction of Failure Region on the Rake Face

The image of rake face contains the wear region, background and cutter body under the point light source, the thresholding techniques that binarize the images are not applicable, instead, a Markov Random Field (MRF) image segmentation method is employed for the failure extraction of the rake face. The MRF model considers the spatial interaction of neighboring pixels; thus, spatial inhomogeneities in the images of the rake face can be processed with this model [45,46,47,48].

Let us assume an image with pixel sites S={(i,j)|1≤i≤N,1≤j≤M}, M×N is the number of pixels of the image. $Y=\left\{y_{ij},\left(i,j\right)\in S\right\}$ represents the observed feature vector. Each pixel (i,j) is assigned a label $v_{ij}$, where $\omega_{ij}\in \Lambda$ represents types of labels. For the rake face, Λ={1,2,3} which corresponds to the failure region, cutter body and background, respectively. The set of the labels $\Omega =\left\{\omega_{ij},\left(i,j\right)\in S\right\}\;$is a random field. The image segmentation is essentially the label process of pixels on the image, the maximum a posteriori which can maximize the posterior probability P(Ω|Y) can be adopted to find out the proper segmentation, as

(12) $${\hat{\omega }}_{ij}^{MAX}=\mathrm{argmax}P\left(\Omega |Y\right)\;$$

**Figure 8:**
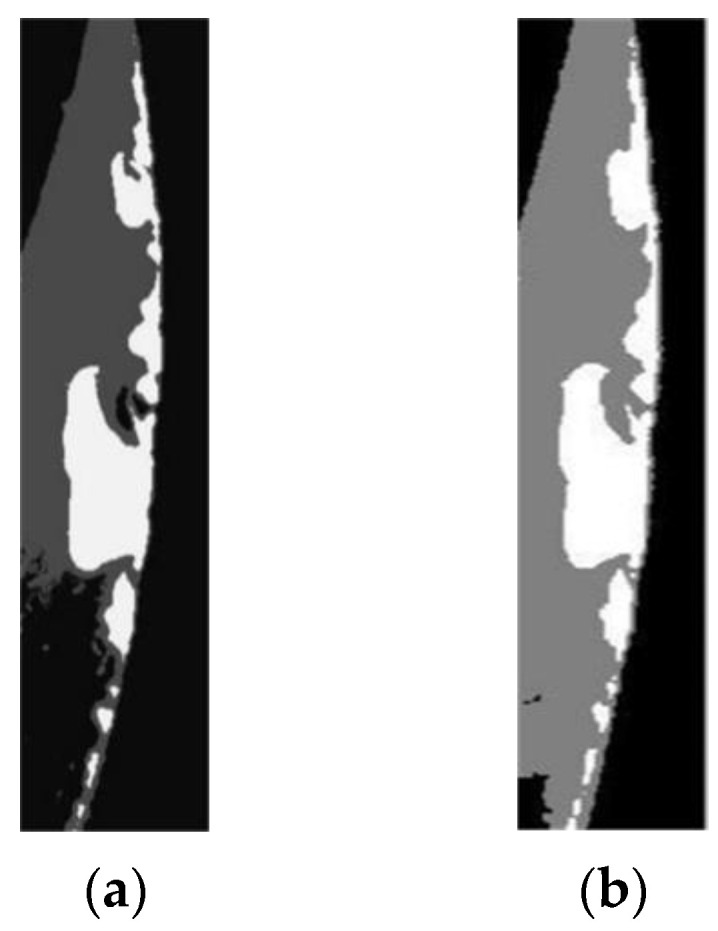
Comparison of the rake face images (**a**) before and (**b**) after segmentation based on MRF model.

Since P(Ω|Y)∝exp(−U(Ω,Y)), Equation (12) can be reduced to

(13) $${\hat{\omega }}_{ij}^{\mathrm{MAX}}=\mathrm{argmin}U\left(\Omega ,Y\right)\;$$

where U(Ω,Y) is the energy function which can be obtained according to Hammersley–Clifford theorem [49]

(14) $$U\left(\Omega ,Y\right)=\underset{S}{\sum }\left(\ln\left(\sqrt{2\pi }\sigma_{\lambda }\right)+\frac{{\left(y_{ij}-\mu_{\lambda }\right)}^{2}}{2\sigma_{\lambda }^{2}}\right)+\underset{i,j}{\sum }\beta \delta \left(\omega_{i},\omega_{j}\right)\;$$

where $\mu_{\lambda }$ and $\sigma_{\lambda }$ are the mean and covariance value of the gray levels of pixels, δ is the Kronecker delta function, β is a weighting parameter that decides the homogeneousness of the region, β is found to have slight effect on the segmentation when its value is larger than 2. Equation (14) is a typical non-convex optimization function, the simulated annealing with Gibbs sampler method can thus be adopted to detect the optimal parameters [50,51]. A comparison between the original preprocessed image and the segmented image of the rake face is shown in Figure 8. The failure region, background and cutter body can be clearly distinguished in the segmented image, the wear region is at the most brightness level. The pixel number of the failure region could hence be calculated easily.

## 4. System Verification

In order to demonstrate the feasibility of the image acquisition system and abovementioned image processing methods, the detection of failure regions of the integral spiral end milling cutters is conducted during the machining process of titanium alloy.

**Figure 9:**
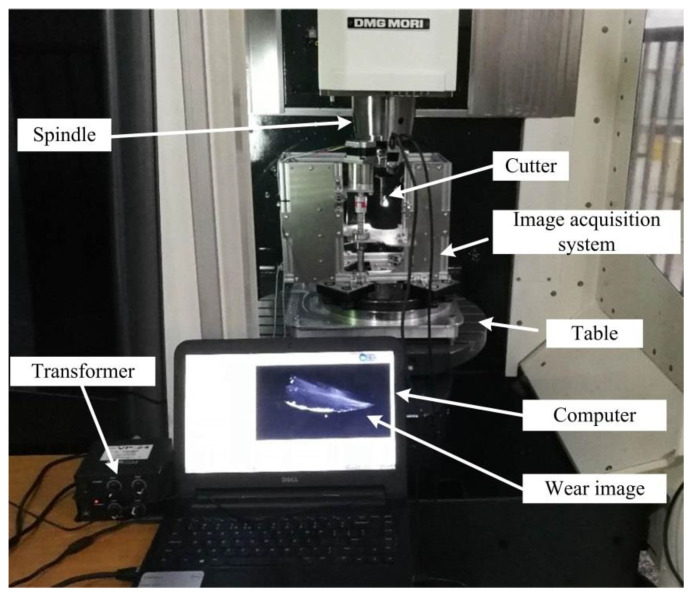
Experiment setup for machining process and tool inspection.

**Figure 10:**
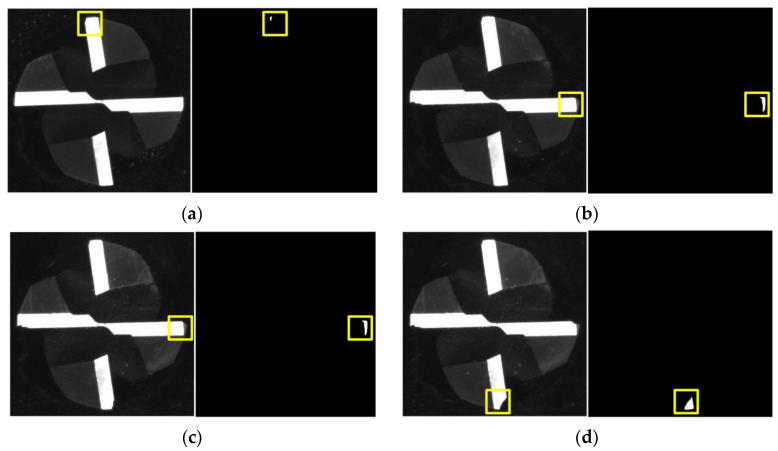
Tool tip status at different time during machining: (**a**) *t* = 3 h; (**b**) *t* = 6 h; (**c**) *t* = 8 h; (**d**) *t* = 9 h. The original unprocessed images of tool tips and extracted largest failure regions are shown in the left and right parts of the subfigures, respectively.

**Figure 11:**
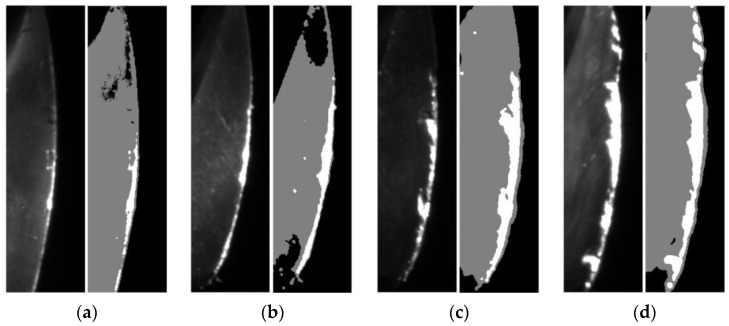
Rake face status at different time during machining: (**a**) *t* = 4 h; (**b**) *t* = 7 h; (**c**) *t* = 10 h; (**d**) *t* = 12 h. The original unprocessed images and processed images with extracted failure regions of the rake face are shown in the left and right parts of the subfigures, respectively.

The experiment setup for machining test and failure detection is shown in Figure 9. The integral spiral end milling cutters with AlCrN coating are employed in the experiment. The diameter of flat end cutters is 16mm. The block workpiece made of titanium alloy TA15M is machined on DMU50 high speed machining center with water-soluble cutting fluid FUCHS ECOCOOL2030S. A face milling process is proposed when the spindle speed is 400 r/min and feed rate per tooth is 0.1 mm. Both the cutting depth and cutting width are set as 2 mm. The images of tool tip and rake face are captured with the image acquisition system at different time during the machining process (Figure 10 and Figure 11). The integrated gas nozzle of the DMG 50 machine tool is employed to blow away the remaining coolant on the cutter when the machining stops in the experiment. The diameter of the air nozzle is about 4mm, and the pressure of compressed air was set as 6 bar.

The different images and extracted failure regions of the integral spiral end milling cutters are given in Figure 10 and Figure 11. It can be seen from Figure 10 and Figure 11 that the coolant is cleared up by the high-pressure air. It should be noted that the failure of the cutter generally occurs on the tool tip and rake face for the machining of titanium alloy, therefore only the failure regions on the tool tip and rake face are given out. The images and failure regions of the tool tip at the 3rd, 6th, 8th and 12th h during the machining process are shown in Figure 10. As shown in Figure 10, there are actually four tool tips in each subfigure and each tool tip contains a different failure region. The proposed detection method could extract the largest failure region as mentioned in Section 3. If the largest failure region on any of the 4 tool tips reaches the failure threshold value, the cutter could be regarded as a failure. The largest failure regions at these machining moments are marked in yellow boxes. The pixel numbers of these regions are 92, 541, 586 and 953, respectively. In addition, the images and failure regions of the rake face at 4th, 7th, 10th, and 12th during the machining process are illustrated in Figure 11. The corresponding pixel numbers of failure regions are 1262, 2242, 3531 and 5253.

It can be concluded from Figure 10 and Figure 11 that the image acquisition system and processing methods have successfully captured the increment in failure regions with the machining time. Besides, the proposed machine vision method could not only detect wear and breakage on the milling cutter, but also enable an estimation of the cutter lifetime with prescribed failure threshold pixel numbers. It is worth mentioning that the failure threshold pixel numbers may vary with types and dimensions of cutters, machining precision and materials and other factors; this could be estimated by an experimental or empirical method, such as statistically analyzing the pixel numbers of failure regions on multiple failed cutters. The milling cutter is considered to reach their life limitation when the failure region pixels numbers on any part of the cutters exceeds their corresponding threshold values and the cutter must then be replaced.

## 5. Conclusions

In this paper, tool wear and breakage detection technologies based on machine vision were proposed for integral spiral end milling cutters. The spatial positions and type of cameras and light sources were designed to develop an image acquisition system which would capture high-quality images of rake face, flank face and tool tip of the milling cutters. Denoising and contrast enhancing were employed for the image preprocessing. Then, the failure regions in images of the flank face and tool tip were extracted after binarization by Otsu thresholding method, while the failure regions in images of the rake face were extracted after segmentation by MRF models. The feasibility of the proposed image acquisition system and image processing methods were eventually examined by the titanium alloy machining process. It was found that the failure regions on the cutters increased with the machining time. The cutters could be regarded as a failure when the failure regions reached their corresponding threshold values. The developed image acquisition system and proposed failure extraction methods provide reliable machine monitoring of the integral spiral end milling cutters during machining processes.

## Data Availability

The data presented in this study are contained within the article.
